# Correction: A tension-adhesion feedback loop in plant epidermis

**DOI:** 10.7554/eLife.57036

**Published:** 2020-03-23

**Authors:** Stéphane Verger, Yuchen Long, Arezki Boudaoud, Olivier Hamant

Verger S, Long Y, Boudaoud A, Hamant O. 2018. A tension-adhesion feedback loop in plant epidermis. *eLife* **7**:e34460. doi: 10.7554/eLife.34460.

Published 12, October 2018

We have been made aware through PubPeer of a mistake in Figure 3 panel F.

Unfortunately this picture has in fact been mistakenly reused from a previous publication and wrongly labeled as being a *qua1-1* mutant while it is in fact a *qua2-1* mutant. This error is partly caused by the similar phenotype of both mutants. As mentioned in the first paragraph of the results:

“The quasimodo1 (qua1) and qua2 mutants, respectively mutated in a galacturonosyltransferase and a pectin methyltransferase, are both required for the synthesis of a fraction of the cell wall pectins. They also display a comparable cell adhesion defect phenotype (Bouton et al., 2002; Mouille et al., 2007). For practical reasons, all the work reported in this study was performed with qua1-1 (WS-4 background), although we observed similar phenotypes in the qua2-1 mutant (Col-0 background)”.

That said, this is no excuse for re-using the same image, and for two different mutants. The first author of the publication worked on figures for both publications (Verger et al., 2016 and Verger et al., 2018) at the same time and mishandled a set of images. None of authors of Verger et al., 2018 noticed it. We now provide an unpublished image of a *qua1-1* mutant in both Figure 3F and Figure 3—figure supplement 1A where they appear, as well as additional images of both mutants and wild type and the corresponding genotypes.

The corrected Figure 3 is shown here:

**Figure 1:**
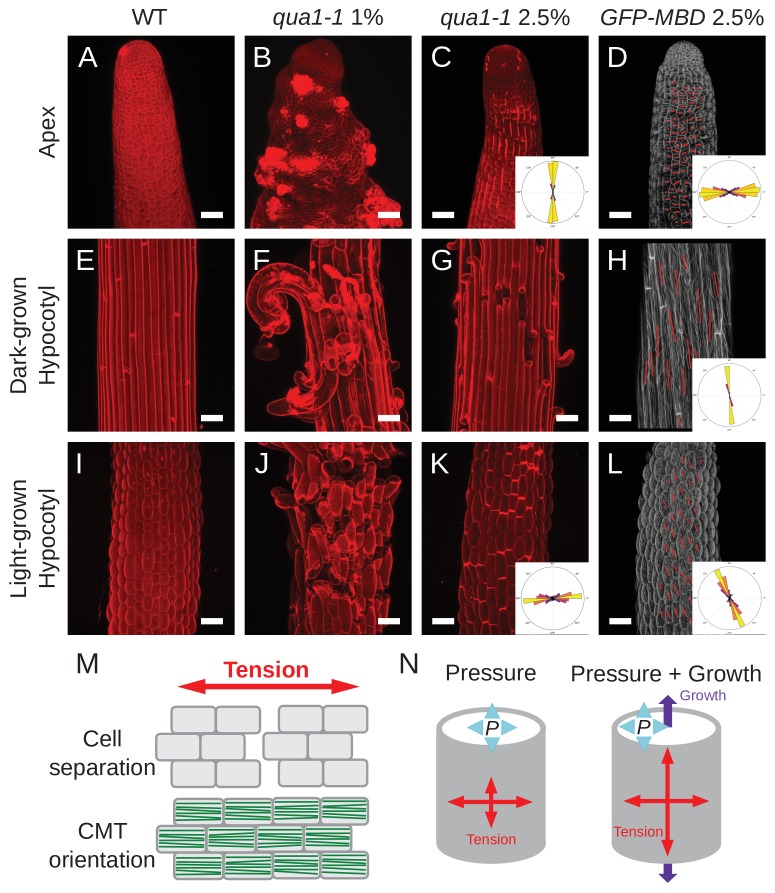


The original Figure 3 is shown here for reference:

**Figure 2:**
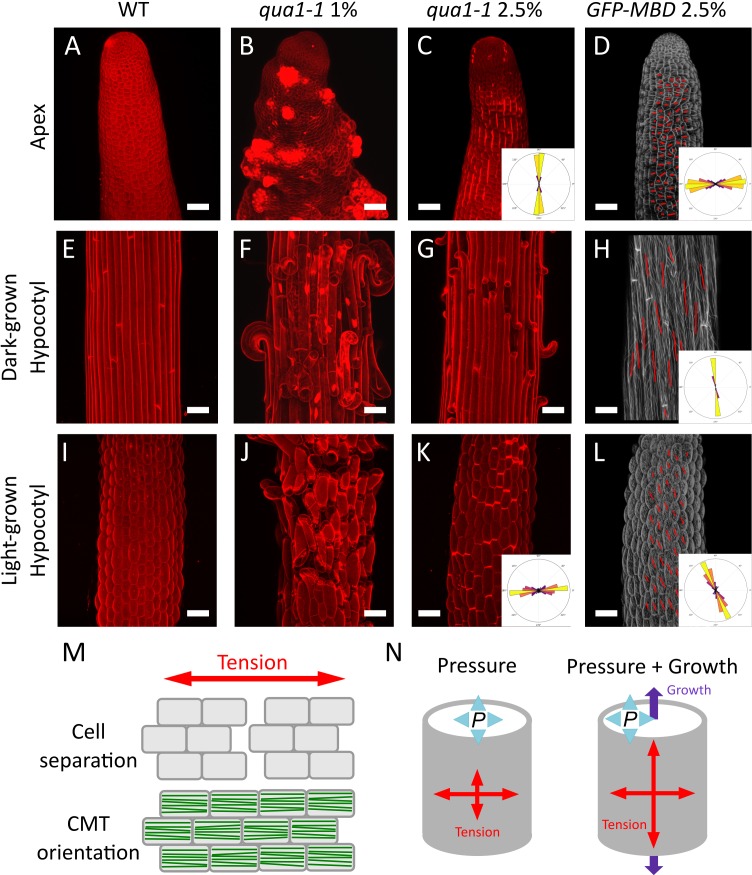


The original panel 3F was also reproduced in Figure 3—figure supplement 1A for convenience. Therefore, we also need to fix Figure 3—figure supplement 1. The corrected Figure 3—figure supplement 1 is shown here:

**Figure 3:**
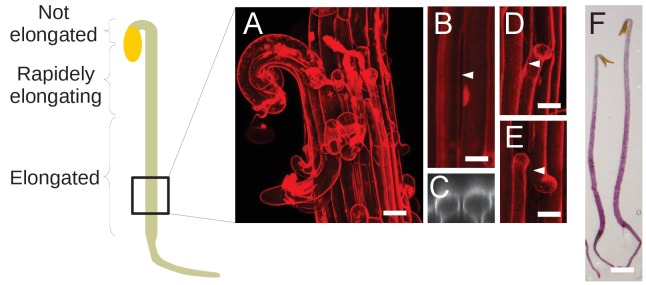


The original Figure 3—figure supplement 1 is shown here for reference:

**Figure 4:**
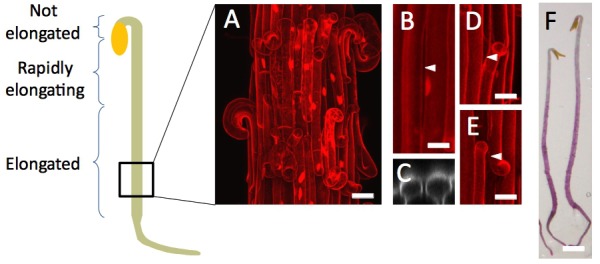


While thoroughly checking the manuscript we also noticed a very minor error in the caption of Figure 3—figure supplement 1: the letter (A) is misplaced. The corrected legend for Figure 3—figure supplement 1 follows (new position of (A) in bold followed by a brief description of the panel):

Dark-grown hypocotyl growth and cell separation patterns.

Schematic representation of a dark-grown hypocotyl, and annotated growth pattern. The square at the bottom represent the relative position where images were taken on dark-grown hypocotyl, in *qua1-1* for cell separation as well as for *GFP-MBD* cortical microtubule orientation. **(A)** Panel reproduced from Figure 3F, for ease of reading. (B) An example of longitudinal cell separation in a dark-grown hypocotyl. (C) Orthogonal view of the cell separation shown in panel B. White arrowhead indicates the site of separation. (D and E) Examples of longitudinal cell separations associated with at least one transverse cell separation. White arrowheads indicate the site of separation. (F) Stereo microscope picture of *qua1-1* dark-grown hypocotyl stained with ruthenium red (a dye that stains unesterified pectins (Steeling, 1970), and thus stains the cell wall only if the cuticle is cracked) revealing more frequent and larger cell separation at the rootward part of the hypocotyl. Scale bar (A), 50 µm. Scale bars (B–E), 20 µm. Scale bar (F), 1 mm.

To clarify this further, we also provide the genotype and phenotype of both *qua1-1* and *qua2-1* (with the corresponding wild-type controls, WS-4 and Col-0).

**Figure 5:**
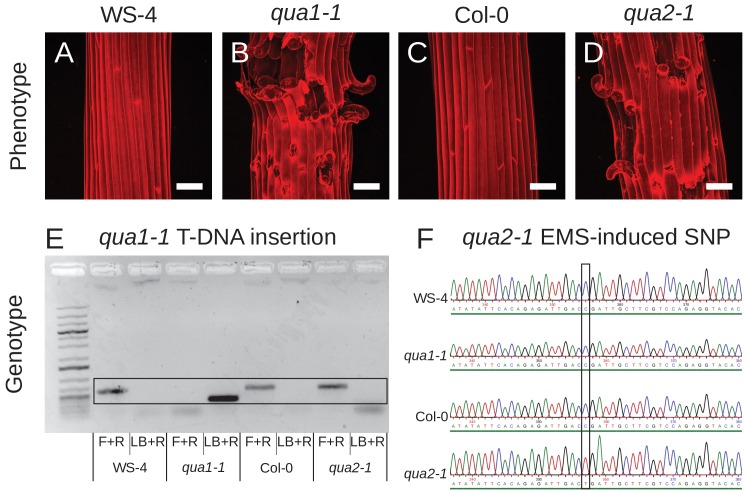


***qua1-1* and *qua2-1* phenotypes and genotypes.**

(**A-D**) Z-projections (maximal intensity) of confocal stacks from representative, propidium iodide stained, dark-grown hypocotyls (WS-4 (**A**), *qua1-1* (**B**), Col-0 (**C**), *qua2-1* (**D**)), showing comparable cell adhesion defects in *qua1-1* and *qua2-1*. Scale bars, 50 µm. (**E**) Genotyping results of the *qua1-1* T-DNA insertion for the four lines. The black frame highlights the band specifically amplified and corresponding to the expected products for the PCR. The wild-type PCR product (F+R) is amplified in WS-4, Col-0 and *qua2-1*, whereas the PCR product corresponding to the T-DNA insertion (LB+R) is amplified in *qua1-1* only. (**F**) Genotyping results of the *qua2-1* EMS-induced SNP for the four lines. The black frame highlights the SNP corresponding to the previously described *qua2-1* mutation at position +1879 of the coding sequence (Mouille et al., 2007). The C to T EMS-induced SNP is only present in the *qua2-1* background.

Plants were grown, prepared and imaged as described in the original publication with the exception of using a Zeiss 800 confocal microscope and a 40x objective instead of 25x for image acquisition.

For genotyping, the following primers were used:

qua1-1_F 5’-CCCAAAATATTTGTCCCGTA-3’

qua1-1_R 5’-TGTGATTCTGCCACCGATTA-3’

Tag_5(LB) 5’-CTACAAATTGCCTTTTCTTATCGAC-3’

qua2-1_F 5’-GAAGATCCATCACCGCCTTA-3’

qua2-1_R 5’-TATGTTTCCGGTTCGGTTTC-3’

For *qua1-1* T-DNA insertion, genotyping was done by PCR amplification followed by gel migration. To identify the presence of the wild-type sequence, the primers qua1-1_F and qua1-1_R (F+R) were used in the PCR mix. To identify the presence of the *qua1-1* T-DNA insertion, the primers Tag_5(LB) (left border of the T-DNA insert) and qua1-1_R (LB+R) were used in the PCR mix.

For *qua2-1* EMS-induced SNP, genotyping was done by PCR amplification followed by sanger sequencing. The primers qua2-1_F and qua2-1_R were used in the PCR mix and the PCR products were sequenced using the qua2-1_F primer.

The corrections do not change any of the scientific conclusions of the manuscript. We apologize for the mistake and the confusion it may have caused.

The article has been corrected accordingly.

