# Supplementary material for: A tension-adhesion feedback loop in plant epidermis
Source: eLife. 2018 Apr 23;7:e34460. doi: 10.7554/eLife.34460 (PMC5963923; doi:10.7554/eLife.34460)
Supplement: Transparent reporting form [file elife-34460-transrepform.docx]

***eLife’s* transparent reporting form**

We encourage authors to provide detailed information *within their submission* to facilitate the interpretation and replication of experiments. Authors can upload supporting documentation to indicate the use of appropriate reporting guidelines for health-related research (see [EQUATOR Network](http://www.equator-network.org/%20)), life science research (see the [BioSharing Information Resource](https://biosharing.org/)), or the [ARRIVE guidelines](http://www.plosbiology.org/article/info:doi/10.1371/journal.pbio.1000412) for reporting work involving animal research. Where applicable, authors should refer to any relevant reporting standards documents in this form.

If you have any questions, please consult our Journal Policies and/or contact us: [editorial@elifesciences.org](mailto:editorial@elifesciences.org).

**Sample-size estimation**

- You should state whether an appropriate sample size was computed when the study was being designed
- You should state the statistical method of sample size computation and any required assumptions
- If no explicit power analysis was used, you should describe how you decided what sample (replicate) size (number) to use

Please outline where this information can be found within the submission (e.g., sections or figure legends), or explain why this information doesn’t apply to your submission:

Sample size and relevant statistical tests have been specified in the main text for all experiments. Details on statistics can also be found in material & methods.

**Replicates**

- You should report how often each experiment was performed
- You should include a definition of biological versus technical replication
- The data obtained should be provided and sufficient information should be provided to indicate the number of independent biological and/or technical replicates
- If you encountered any outliers, you should describe how these were handled
- Criteria for exclusion/inclusion of data should be clearly stated
- High-throughput sequence data should be uploaded before submission, with a private link for reviewers provided (these are available from both GEO and ArrayExpress)

Please outline where this information can be found within the submission (e.g., sections or figure legends), or explain why this information doesn’t apply to your submission:

- Biological replicates are similar experiments done at different days, from multiple independent equivalent samples. Technical replicates are similar experiments done several times, the same day, on the same sample.

- All experiments have been performed as 3 biological replicates, and at least 3 samples per genotype/condition were assayed for each biological replicate, as mentioned in material & methods.

- Atomic Force microscopy experiments include at least 9 technical replicates for each assayed cell, and 3 technical replicates at each indentation position, as mentioned in material & methods.

- All acquired data were quantified as described in material & methods. Note that Propidium Iodide staining (to reveal cell separation) sometimes produced images that were not suitable for our image analysis pipeline. Specifically when the background signal between separated cells was too high and sometimes at similar levels compared to the cells signal, making it close to impossible to properly segment the cell separations. To avoid segmentation errors, these images were excluded.

**Statistical reporting**

- Statistical analysis methods should be described and justified
- Raw data should be presented in figures whenever informative to do so (typically when N per group is less than 10)
- For each experiment, you should identify the statistical tests used, exact values of N, definitions of center, methods of multiple test correction, and dispersion and precision measures (e.g., mean, median, SD, SEM, confidence intervals; and, for the major substantive results, a measure of effect size (e.g., Pearson's r, Cohen's d)
- Report exact p-values wherever possible alongside the summary statistics and 95% confidence intervals. These should be reported for all key questions and not only when the p-value is less than 0.05.

Please outline where this information can be found within the submission (e.g., sections or figure legends), or explain why this information doesn’t apply to your submission:

Different statistical tests have been performed to compare averages and distributions, as presented in material & methods and in the main text. In all cases, the type of statistical test, the sample size, the mean, the SD and the p-value are reported along with each described experiments.

Here is a excerpt from material & methods:

For linear data (comparing gap area, anisotropy), classical statistical tests were used. Normality of the samples was tested using Shapiro’s test. If at least one of the sample population did not have a normal distribution, the populations were compared with the non parametric Wilcoxon Rank Sum test. If both samples had normal distribution, their variance were compared using Bartlett’s test. If they were equal, a Student’s *t*-test was performed; if they were unequal, a Welch’s *t*-test was performed.

For circular (or directional) data (cell separation orientation and CMT orientation) we used circular statistics. Most of our sample did not follow a Von Mises distribution, thus only non-parametric tests were used. The Rao’s spacing test was used to determine if the populations of angles were homogeneously distributed, or had a preferred orientation determined by the circular mean.

Finally the data form the CMT response to ablation were treated as linear data, since the calculated angle was between 0° and 90°. The symmetry of CMT response distribution was measured by the skewness of the population towards 90° and the significance of the skewness was tested against a normal distribution.

(For large datasets, or papers with a very large number of statistical tests, you may upload a single table file with tests, Ns, etc., with reference to sections in the manuscript.)

**Group allocation**

- Indicate how samples were allocated into experimental groups (in the case of clinical studies, please specify allocation to treatment method); if randomization was used, please also state if restricted randomization was applied
- Indicate if masking was used during group allocation, data collection and/or data analysis

Please outline where this information can be found within the submission (e.g., sections or figure legends), or explain why this information doesn’t apply to your submission:

Groups were defined genetically (typically WT vs. mutant) or experimentally (control vs. treated) as mentioned in the main text and figure legends.

**Additional data files (“source data”)**

- We encourage you to upload relevant additional data files, such as numerical data that are represented as a graph in a figure, or as a summary table
- Where provided, these should be in the most useful format, and they can be uploaded as “Source data” files linked to a main figure or table
- Include model definition files including the full list of parameters used
- Include code used for data analysis (e.g., R, MatLab)
- Avoid stating that data files are “available upon request”

Please indicate the figures or tables for which source data files have been provided:

This study does not involve very large datasets, thus no additional data file has been uploaded. Mathematical methods have been described in the material and method section. We have uploaded the annotated python script that we developed to perform the cell separation analysis.
